# Are Auditory Steady-State Responses Useful to Evaluate Severe-to-Profound Hearing Loss in Children?

**DOI:** 10.1155/2015/579206

**Published:** 2015-10-18

**Authors:** Signe Schuster Grasel, Edigar Rezende de Almeida, Roberto Miquelino de Oliveira Beck, Maria Valéria Schmidt Goffi-Gomez, Henrique Faria Ramos, Amanda Costa Rossi, Robinson Koji Tsuji, Ricardo Ferreira Bento, Rubens de Brito

**Affiliations:** Department of Otolaryngology, University of São Paulo, 255 Dr. Eneas de Carvalho Aguiar Avenue, 05403-900 São Paulo, SP, Brazil

## Abstract

*Objective*. To evaluate Auditory Steady-State Responses (ASSR) at high intensities in pediatric cochlear implant candidates and to compare the results to behavioral tests responses. *Methods*. This prospective study evaluated 42 children with suspected severe-to-profound hearing loss, aged from 3 to 72 months. All had absent ABR and OAE responses. ASSR were evoked using binaural single frequency stimuli at 110 dB HL with a 10 dB down-seeking procedure. ASSR and behavioral test results were compared. *Results*. Forty-two subjects completed both ASSR and behavioral evaluation. Eleven children (26.2%) had bilateral responses. Four (9.5%) showed unilateral responses in at least two frequencies, all confirmed by behavioral results. Overall 61 ASSR responses were obtained, most (37.7%) in 500 Hz. Mean thresholds were between 101.3 and 104.2 dB HL. Among 27 subjects with absent ASSR, fifteen had no behavioral responses. Seven subjects showed behavioral responses with absent ASSR responses. No spurious ASSR responses were observed at 100 or 110 dB HL. *Conclusion*. ASSR is a valuable tool to detect residual hearing. No false-positive ASSR results were observed among 42 children, but in seven cases with absent ASSR, the test underestimated residual hearing as compared to the behavioral responses.

## 1. Introduction

As universal newborn hearing screening programs are established in numerous countries, more children will be diagnosed in early childhood with some degree of hearing loss.

Early detection and intervention during the critical period for language and cognitive development can improve individual performance [1].

Children with severe-to-profound bilateral hearing loss are candidates for cochlear implantation (CI) and require specific audiologic evaluation prior to intervention. As early age of indication and presence of residual hearing are important factors for postimplant speech perception and language development, this has resulted in further decrease of minimum age of surgery [2–7].

In these very young children, behavioral audiologic evaluation can be challenging, may not be obtained in children younger than 6 months, and usually does not assess each ear separately. Thus the audiologic evaluation of pediatric cochlear implant candidates relies more and more on electrophysiological measures.

The most widely used electrophysiological procedure for estimating hearing thresholds in young children is click and tone burst auditory brainstem responses (ABR). Due to the transient nature of the stimuli used to evoke ABR, maximum output levels are 95 dB hearing level (HL). In view of that, the possibility of residual hearing at severe-to-profound levels cannot be investigated with ABR [8].

Hearing assessment of children, using the Auditory Steady-State Responses (ASSR), is made by frequency specific continuous modulated tones and allows increased levels of stimulation intensity. Therefore, ASSR can provide ear and frequency specific threshold information at elevated intensity levels up to 120 dB HL and higher, providing better and more reliable investigation of ears with minimal residual hearing [9]. Furthermore, ASSR thresholds may be used for hearing aid fitting prior to cochlear implantation.

For such reasons, ASSR is a unique tool for auditory assessment of young cochlear implant candidates.

Some authors [10, 11] have investigated the use of ASSR to evaluate patients with severe-to-profound hearing loss. They showed that spurious responses might occur during high stimulus intensities, especially in 500 and 1000 Hz.

Solutions have been implemented by the manufacturer to reduce artifacts at high-intensity stimulation [12].

Few papers have been published since 2004. One report evaluated 15 children with severe-to-profound hearing loss by ASSR, but behavioral thresholds were obtained for only one subject [13]. As cochlear implant is the first choice, especially, for the young child with severe-to-profound hearing loss, it is quite important to obtain more data in the pediatric population.

Previously, we performed two studies at the University of São Paulo. One of them evaluated adults with severe-to-profound hearing loss. The responses of pure tone audiometry (PTA) and ASSR were compared. Patients' subjective perception of ASSR stimuli was also evaluated and compared to PTA test results, and no systematic extra-auditory ASSR responses at high intensities were observed [14].

The other study evaluated children with severe-to-profound hearing loss from 6 to 65 months. Most ASSR responses (48%) were found at 500 Hz [15].

The aim of this study was to evaluate Auditory Steady-State Responses (ASSR) at high intensities in pediatric cochlear implant candidates and to compare the results to behavioral test responses.

## 2. Materials and Methods

### 2.1. Subjects

This prospective study evaluated 58 children with suspected severe-to-profound hearing loss, aged from 3 to 72 months. All children referred to this institution for pediatric cochlear implant evaluation between January and December 2011 were enrolled.

We* included* only children with normal external and middle ear conditions.

We* excluded* patients with severe neurologic disorders who did not permit behavioral evaluation. We also excluded patients who showed responses on either ABR or OAE or who did not achieve noise ratio under 30 microvolts during ASSR.

Overall 16 patients were excluded: three had severe neurologic disorders and could not complete behavioral evaluation, one showed responses on click ABR, one had bilateral absent click ABR and normal DPOAE (possible auditory neuropathy spectrum disorder), one showed high noise levels during ASSR, and ten did not show up for behavioral evaluation.

### 2.2. Methods

The procedure was a routine assessment for pediatric cochlear implantation at the Department of Otolaryngology, University of São Paulo School of Medicine. The study was reviewed and approved by the Hospital's Ethic Committee (number 38954) and written informed consent was obtained from all parents.

In a unique session using light general anesthesia with Sevoflurane, all children were examined by otomicroscopy and tympanometry followed by click auditory brainstem responses (click ABR), bone conduction ABR, distortion-product otoacoustic emissions (DPOAE), and ASSR.

All ABR, OAE, and ASSR recordings were obtained in a sound-treated room.

### 2.3. Stimuli and Recordings

#### 2.3.1. ABR and Otoacoustic Emissions

The tests were run using the Navigator Pro SCOUT and AEP (Natus Bio-Logic Systems Corp., Mundelein, IL) software.

EEG activity was recorded using gold disk electrodes placed on the earlobe and Fpz. The contralateral earlobe was used as ground. Interelectrode impedance was less than 2 KOhm.

Click stimuli (duration: 100 milliseconds) were presented with ER3A insert earphones at the maximum level of 90 dB HL at a rate of 21.1/s with rarefaction and condensation polarity. The responses were considered to be absent when both rarefaction and condensation waves showed no responses at 90 dBHL. Bone conduction was tested with a standard bone vibrator at the maximum intensity of 55 dB HL using alternated click stimuli and contralateral masking of the same intensity.

For DPOAE we applied the diagnostic 750 to 8000 Hz test protocol (Navigator Pro SCOUT, Natus Bio-Logic Systems Corp., Mundelein, IL). Responses greater than 6 dB over background noise (signal-to-noise ratio) at five out of eight frequencies were considered as Pass criteria.

#### 2.3.2. ASSR

The ASSR test was completed in the same session as ABR and OAE. ASSR were evoked using binaural single frequency stimuli at 110 dBHL with a 10 dB down-seeking procedure. Test stimuli were 0.5, 1, 2, and 4 kHz tones modulated in amplitude and frequency. Stimuli were 20% frequency and 100% amplitude modulated at 65 Hz for all tones in the left ear and at 69 Hz for all tones in the right ear according to the default specifications of the ASSR system (Navigator Pro MASTER, Natus Bio-Logic Systems Corp., Mundelein, IL). Modulation rates of 65 Hz or higher were used to ensure acceptable signal-to-noise ratio for response detection. Test stimuli were presented through insert earphones (ER3A) previously calibrated for each frequency as suggested by the manufacturer. Measurements were made with a Brüel & Kjaer sound level meter DB-0138 which conforms to ANSI S3.7-1995.

The ASSR assessments were performed by a dichotic single frequency technique. This implies that a single frequency was offered to both ears simultaneously. Electrode disks were fixed with electrolytic paste to the scalp at Cz (active), midline posterior neck (reference), and Fpz (ground). All electrode impedances were below 5 KOhm, and the interelectrode impedance values were kept below 3 KOhm. A maximum of 10 sweeps containing 16 epochs each were recorded per trial. Each epoch was 1.024 seconds. The electrophysiological recording was converted by means of a fast Fourier transform after each sweep. The response was accepted with an F ratio, comparing the fast Fourier components at the stimulus modulation frequencies to determine whether the difference was significantly different from the background noise (*P* < 0.05). If a sweep contained more than 80 nV of electrophysiological noise, it was rejected. Thresholds were repeated to guarantee reliable results, completing 10 sweeps in each run. The same physicians performed all tests.

#### 2.3.3. Behavioral Evaluation

Two experienced audiologists did the behavioral evaluation in a double-walled sound booth in a free field test condition. Children were tested in one or more sessions. We chose instruments because 3-month-old infants would not be able to perform visual reinforcement audiometry.

The stimuli were uncalibrated sounds, presented at low 55–80 dB SPL, medium 70–88 SPL, and high intensities 80–115 dB SPL, depending on the instrument. Three frequency ranges were evaluated. We used drums for the low frequency spectrum (125–500 Hz), wooden rattle and* agogo* (Brazilian instrument) for mid-low frequencies (1000–3000 Hz), and metallic rattle and bell for high frequencies (over 3000 Hz) [16, 17]. A response was considered positive if the subject localized the stimulus in a lateral, superior, or inferior plane, according to Dworsack-Dodge et al. [18], in one or more frequency ranges. We considered a positive response, if the child changed the suction behavior, increased or decreased facial or body movements, or started crying or showed a startle reflex time-locked to the stimulus [18].

Children had behavioral evaluation within three months of the electrophysiological test battery.

#### 2.3.4. Criteria for Data Evaluation

We analyzed four ASSR frequencies in each ear (500, 1000, 2000, and 4000 Hz). The percentage of present or absent responses at each frequency was determined. We considered present ASSR responses, if responses could be obtained in at least two frequencies in one or both sides.

For the behavioral test, we evaluated if the participant had present or absent responses to the frequency range of low, mid-low, and high frequencies. No ear specific responses were obtained in this test condition.

#### 2.3.5. Comparison between Both Tests

ASSR and behavioral test results were compared, regarding present or absent responses. Therefore, we divided the subjects into four groups.

(1) Present responses in both tests.
(2) Absent responses in both tests.
(3) Present responses in ASSR and no responses in behavioral evaluation.
(4) No responses in ASSR and present responses in behavioral evaluation.

### 2.4. Statistical Analysis

Association between ASSR and behavioral test results was evaluated by Kendall's rank correlation tau and Cohen's kappa coefficient. The software used was “R statistical computing.”

## 3. Results

From 58 children enrolled in the study, forty-two completed both ASSR and behavioral evaluation.

After all we studied 42 subjects (20 girls and 22 boys) between 6 and 60 months (mean age: 29.3 months, median age: 26.0 months, and SD: 15.6 months).

**Figure 1:**
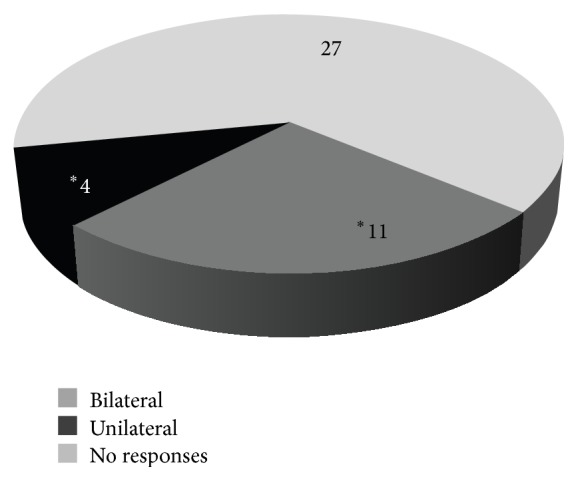
ASSR responses (*n* = 42 subjects). Unilateral and bilateral responses were considered only if they were positive in at least 2 frequencies.

Fifteen subjects (35.7%) showed ASSR responses in two or more frequencies. Eleven had bilateral and 4 had unilateral responses (Figure 1).

Most responses (37.7%) were obtained at 500 Hz, 29.5% at 1000 Hz, 21.3% at 2000 Hz, and 11.4% at 4000 Hz (Table 1).

**Figure 2:**
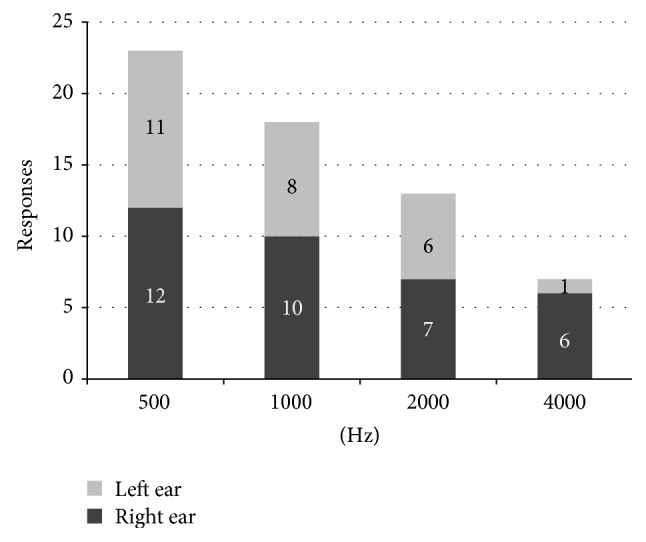
Right and left ear ASSR responses.

**Figure 3:**
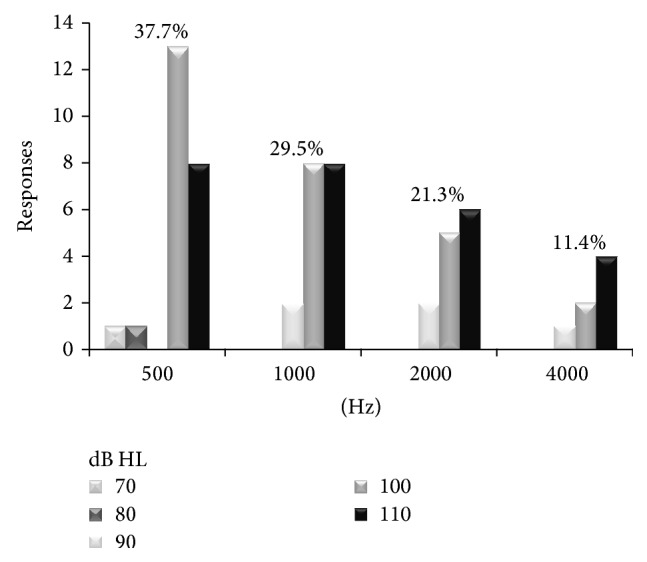
ASSR thresholds for each frequency (*n* = 61 responses).

Right and left ear responses are presented in Figure 2. Frequency specific thresholds are shown in Figure 3. Twenty-seven subjects (64.3%) had absent ASSR responses (Table 2).

At behavioral evaluation we found 34 responses (48.6%) in the low frequency, 24 (34.3%) in mid-low frequency, and 12 (17.1%) in the high frequency range. Twenty-four children had responses to more than one instrument. Seven subjects of 42 (16.6%) had absent responses to all frequency ranges.

### 3.1. Comparison between ASSR and Behavioral Test Results

**Figure 4:**
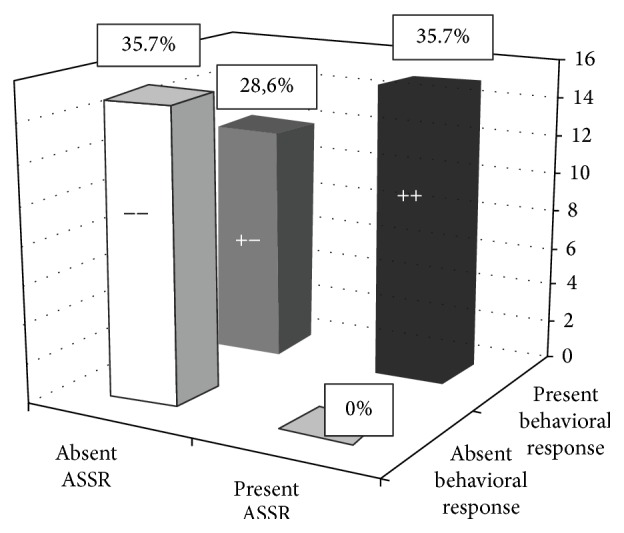
ASSR and behavioral tests correlated well in 71.4% of the subjects: 15 children had present responses in both tests and 15 had absent responses in both tests.

Fifteen subjects had responses in both tests (group 1), fifteen had absent responses in both tests (group 2), no subject had present ASSR and absent behavioral responses (group 3), and twelve had absent responses in ASSR and present responses at the behavioral test (group 4) (Figure 4).

In group 4, five subjects had single frequency responses on ASSR (two at 500 Hz, two at 1000 Hz, and one at 2000 Hz) but were considered nonresponders for data analysis.

Overall, in 30 subjects (71.4%), both tests showed consistent results.

## 4. Discussion

Accurate diagnosis of a severe-to-profound bilateral sensorineural hearing loss remains the primary and most basic requirement for implantation [19]. The ASSR may therefore assist in the decision of cochlear implant candidacy in young infants in whom specific audiologic challenges related to the limitations of the audiometric test battery are encountered [20].

The possibility of an objective evaluation at this age is very important for the correct decision of cochlear implantation and of the side to be implanted, in case of a unilateral CI. Still, behavioral responses should be obtained and correlated with ASSR responses.

Our results demonstrate a strong correlation between ASSR and behavioral test results in 71.4% of the subjects. Moreover, no false-positive ASSR responses were seen in this casuistic, differing from other authors [10, 11].

In this study we excluded patients with external and middle ear disorders, because the focus was on pure severe-to-profound sensorineural hearing loss. Children with suspected auditory neuropathy spectrum disorder (ANSD) or neurologic disabilities were also excluded, as behavioral testing may be very difficult or even unreliable in some cases. Previous reports showed that ASSR thresholds in ANSD may be substantially higher than pure tone thresholds [21], so currently the value of ASSR in evaluating ANSD is still controversial.

In all frequency range both tests showed consistent results, as subjects responded best to low frequencies and less in the high frequency range. Since instruments used for the behavioral test are not frequency specific but encompass a wider frequency range, it is not surprising to observe higher number of responses in this test. This is especially true for the low frequency range, because 250 Hz was not tested during ASSR, but may have contributed to the higher number of behavioral responses in this frequency range (125 to 500 Hz).

Stimulation of the low frequency range in high intensities may evoke responses due to vibration and may not reflect the auditory status [9]. So caution is recommended when considering responses in this frequency range at high intensities.

All subjects with absent behavioral responses had no responses in ASSR. In other words, no subject had spurious or extra-auditory ASSR responses in our cohort, differing from other studies [10, 11]. Few studies have been published comparing high-intensity ASSR results to behavioral tests in the pediatric population [13, 22]. Swanepoel and Hugo [13] evaluated 15 children with severe-to-profound hearing loss by ASSR, but behavioral thresholds were obtained for only one subject. The same group [22] evaluated 10 children (between 10 and 15 years) with severe-to-profound hearing loss. They found no significant difference between ASSR and behavioral thresholds, except at 500 Hz. This small casuistic (*N* = 10) was composed of older children (mean age: 13 years and 4 months), old enough to perform pure tone audiometry. These results are not comparable to the present study with younger patients (mean age: 2 years and 5 months) who were evaluated by an instrument-based behavioral test, since visual reinforcement audiometry is not a test tool for infants aged 3 months. It seems that ASSR has been unpopular for evaluating pediatric cochlear implant candidates. A reason may be that not all equipment permits ASSR stimulation above 100 dB HL, as it is the case for newer equipment, using the Chirp stimulus for ASSR assessment. To our knowledge, there are no published data about ASSR threshold obtained with the Chirp stimulus in high-intensity levels.

Nevertheless, high-intensity ASSR is a promising tool to evaluate residual hearing in children with severe-to-profound hearing loss and absent responses to click ABR. The continuous amplitude and frequency-modulated tones make it possible to determine frequency specific thresholds at high intensities by means of an objective evaluation, with minimal interference of the examiner. Thus, it is a unique electrophysiological technique to get frequency specific thresholds at intensities exceeding 95 dB HL, where tone ABR rarely will detect responses, due to the transient nature of the tone burst stimulus. Moreover, tone ABR depends on visual wave identification by the clinician who should be well trained and cautious.

As ear specific information is available, ASSR thresholds can be used for hearing aid fitting before eventual cochlear implantation. Furthermore, in case of unilateral responses, four of 42 subjects in this study, the results may assist the surgical team to choose the side to be implanted, if unilateral CI is the option.

As in other reports [22, 23], most children exhibited bilateral ASSR responses.

The Navigator Pro MASTER software permits simultaneous stimulation of both ears at one test frequency even at high intensities (>80 dB HL), so overall test time is reduced. Although test time was not the scope of our study, the whole procedure (otomicroscopy/tympanometry and the electrophysiological test battery including click ABR, bone conduction ABR, DPOAE, and ASSR) did not exceed 60 minutes per patient. In our institution these tests are performed by the same physicians for more than four years and are part of routine evaluation before pediatric cochlear implantation, so this may have contributed to the acceptable overall test time.

We considered present ASSR responses, if they could be obtained in at least two frequencies in one or both sides to prevent spurious or extra-auditory responses at single frequencies. However, this decision turned ASSR less sensitive than the behavioral evaluation, where one instrument encompasses a wide acoustic spectrum and stimulates a range of frequencies.

Unlike Swanepoel and Hugo [13] we did not exceed the stimulus intensity of 110 dB HL in any frequency, so we may have missed some ASSR responses at very high intensities. This might have caused twelve subjects in our study to show behavioral responses with absent ASSR, as defined by our criteria. Among these subjects, five had single frequency responses, not considered auditory responses in our study, perhaps underestimating residual thresholds. But seven children had behavioral responses in more than one frequency range with no response at all in ASSR. In these cases, ASSR probably underestimated auditory thresholds. These results differ from other studies [14, 22] obtained in older children and adults. The response amplitude in children is usually smaller, so the signal-to noise ratio is poorer in the very young age group [24]. Responses may be difficult to be detected even at normal hearing levels, as response amplitude decreases toward threshold [25]. So the clinician should be cautious and not overestimate ASSR when testing young children. At least at 110 dB HL, absent ASSR may not predict absent behavioral responses in all children. We do not recommend including higher intensities (120 dB or higher) in routine evaluation of pediatric cochlear implant candidates, because these very high intensities may damage the cochlea and the clinician may be unaware of behavioral test results, as the electrophysiological tests usually precede the behavioral tests.

Therefore, a complete test battery including electrophysiological and behavioral measures is still the best means to correctly evaluate hearing thresholds, even in cases of severe-to-profound hearing loss.

## 5. Conclusion

ASSR is a valuable tool to detect residual hearing in young children with absent ABR and DPOAE. No false-positive ASSR results were observed among 42 subjects, but in seven cases with absent ASSR, the test underestimated residual hearing as compared to the behavioral responses.

## Figures and Tables

**Table 1:** Percentage of ASSR responses for each frequency (mean thresholds and standard deviation).

Frequency	Responses (%)	Mean/SD
500 Hz	37.7	101.3/±9.6
1000 Hz	29.5	103.3/±6.8
2000 Hz	21.3	103.0/±7.5
4000 Hz	11.4	104.2/±7.8

**Table 2:** ASSR responses (*n* = 42 subjects), thresholds in dB HL.

Patients	Age	500 Hz		1000 Hz		2000 Hz		4000 Hz	
	(months)	R	L	R	L	R	L	R	L
1	8	NR	100	NR	NR	NR	NR	NR	NR
2	11	NR	NR	NR	NR	100	NR	NR	NR
3	31	100	100	100	100	100	100	100	NR
4	28	NR	NR	NR	NR	NR	NR	NR	NR
5	60	NR	NR	NR	NR	NR	NR	NR	NR
6	22	NR	100	NR	NR	NR	NR	NR	NR
7	23	NR	NR	NR	NR	NR	NR	NR	NR
8	27	110	NR	NR	NR	NR	NR	NR	NR
9	47	NR	NR	NR	NR	NR	NR	NR	NR
10	24	NR	NR	NR	NR	NR	NR	NR	NR
11	48	NR	NR	NR	NR	NR	NR	NR	NR
12	53	NR	NR	NR	NR	NR	NR	NR	NR
13	29	110	NR	110	NR	110	NR	110	NR
14	36	NR	NR	NR	NR	NR	NR	NR	NR
15	34	NR	NR	NR	NR	NR	NR	NR	NR
16	23	NR	NR	NR	NR	NR	NR	NR	NR
17	30	NR	NR	NR	100	NR	NR	NR	NR
18	21	NR	NR	NR	NR	NR	NR	NR	NR
19	36	NR	NR	NR	NR	NR	NR	NR	NR
20	17	70	NR	90	NR	90	NR	100	NR
21	6	100	NR	NR	110	NR	NR	NR	NR
22	21	NR	NR	NR	100	NR	NR	NR	NR
23	12	NR	NR	100	NR	NR	NR	NR	NR
24	23	80	NR	100	NR	NR	NR	NR	NR
25	9	NR	110	110	110	NR	110	NR	110
26	25	NR	NR	NR	NR	NR	NR	NR	NR
27	53	100	100	NR	NR	NR	NR	NR	NR
28	48	100	100	90	100	100	90	NR	NR
29	12	NR	NR	NR	NR	NR	NR	NR	NR
30	13	NR	NR	NR	NR	NR	NR	NR	NR
31	36	NR	NR	NR	NR	NR	NR	NR	NR
32	57	110	110	110	NR	110	110	110	NR
33	39	NR	NR	NR	NR	NR	110	NR	NR
34	24	NR	100	NR	NR	NR	NR	NR	NR
35	60	110	100	110	110	110	100	NR	NR
36	13	100	100	NR	NR	NR	NR	NR	NR
37	34	NR	NR	NR	100	NR	NR	90	NR
38	36	NR	NR	NR	NR	NR	NR	NR	NR
39	57	NR	NR	NR	NR	NR	NR	NR	NR
40	14	NR	NR	NR	NR	NR	NR	NR	NR
41	9	NR	NR	NR	NR	NR	NR	NR	NR
42	22	110	110	110	NR	NR	NR	110	NR

R = right ear; L = left ear; NR = no response.
